# Effect of cogon grass root ethanol extract on fatty acid binding protein 4 and oxidative stress markers in a sepsis mouse model

**DOI:** 10.12688/f1000research.73561.3

**Published:** 2023-10-03

**Authors:** Mirasari Putri, Bening Mauliddina Rastiarsa, Raden Aliya T. M. Djajanagara, Ghaliby Ardhia Ramli, Neni Anggraeni, Nugraha Sutadipura, Nur Atik, Mas Rizky A. A. Syamsunarno

**Affiliations:** 1 Department of Biochemistry, Nutrition and Biomolecular, Faculty of medicine. Universitas Islam Bandung, Bandung, West-Java, 40616, Indonesia; 2 Faculty of Medicine, Universitas Padjadjaran, Sumedang, West Java, 45363, Indonesia; 3 Faculty of Medicine, Universitas Islam Bandung, Bandung, West-Java, 40616, Indonesia; 4 Medical Laboratorium Technologist, Bakti Asih School of Analyst, Bandung, West-Java, 40192, Indonesia; 5 Department of Biomedicine Sciences, Faculty of Medicine, Universitas Padjadjaran, West Java, 45363, Indonesia

**Keywords:** Cogon grass, FABP4, sepsis, oxidative stress, inflammation

## Abstract

**Background:** Sepsis causes several immunological and metabolic alterations that induce oxidative stress. The modulation of fatty acid-binding protein 4 (FABP4) has been shown to worsen this condition. Extract of cogon grass root (ECGR) contains flavonoids and isoeugenol compounds that exhibit anti-inflammatory and antioxidant properties. This study aimed to assess the effects of ECGR on FABP4 and oxidative stress–related factors in a sepsis mouse model.

**Methods:** Twenty-nine male mice ( *Mus musculus*) of the *Deutsche Denken Yoken* strain were divided into four groups: group 1, control; group 2, mice treated with 10 μL/kg body weight (BW) lipopolysaccharide (LPS); and groups 3 and 4, mice pre-treated with 90 and 115 mg/kg BW, respectively, and then treated with 10 μL/kg BW LPS for 14 d. Blood, liver, lymph, and cardiac tissue samples were collected and subjected to histological and complete blood examinations. Antioxidant (Glutathione peroxidase 3 (GPx3) and superoxide dismutase), FABP4 levels, and immune system-associated biomarker levels (TNF-α, IL-6 and IL-1β) were measured.

**Results:** Significant increases in platelet levels (p = 0.03), cardiomyocyte counts (p =0.004), and hepatocyte counts (p = 0.0004) were observed in group 4 compared with those in group 2. Conversely, compared with those in group 2, there were significant decreases in TNF-α expression in group 3 (p = 0.004), white pulp length and width in group 4 (p = 0.001), FABP4 levels in groups 3 and 4 (p = 0.015 and p = 0.012, respectively), lymphocyte counts in group 4 (p = 0.009), and monocyte counts (p = 0.000) and polymorphonuclear cell counts in the livers (p = 0.000) and hearts (p = 0.000) of groups 3 and 4. Gpx3 activity was significantly higher in group 3 than in group 1 (p = 0.04).

**Conclusions:** ECGR reduces FABP4 level and modulating oxidative stress markers in sepsis mouse model.

## Introduction

Sepsis significantly contributes to morbidity, mortality, and healthcare expenditure worldwide, with approximately 20 million cases of sepsis occurring each year. ^1^ According to the 2015 International Multicenter Prevalence Study on Sepsis (IMPRESS STUDY), Asia has the highest global sepsis morbidity rate. ^2^ Sepsis is a life-threatening condition characterized by unregulated systemic inflammation and oxidative responses to infection that can cause organ damage. ^3^ Sepsis involves several molecular mechanisms of inflammation and cell damage, including the release of cytokines, eicosanoids, and free radicals. ^4^ Specifically, free radicals may mediate cell damage and contribute to the development of liver, spleen, and heart failure, ^5^^–^^7^ resulting in multiple organ failure (MOF) and mortality. ^8^ In mammals, glutathione peroxidase (GPx) and superoxide dismutase (SOD) are the main antioxidants that protect cells from damage caused by free radicals through synergistic action. ^9^^,^^10^

In addition to the immunologic response and oxidative stress, various metabolic alterations also occur in sepsis. Serious infections cause increased lipolysis of adipose tissue, allowing free fatty acids (FFAs) to be used for triglyceride synthesis in the liver. ^11^^,^^12^ This phenomenon aligns with the high levels of triglycerides and FFAs found in patients in sepsis, which are associated with reduced hepatic fatty acid oxidation. ^12^^,^^13^ Specifically, cytosolic fatty acid-binding protein 4 (FABP4, or adipocyte protein 2) is a lipid chaperone that regulates lipid transport in adipocytes and macrophages. ^14^ Recent studies have found that FABP4 increases the severity of inflammation-related diseases by elevating the expression of cytokines, such as TNF-α, IL-1, IL-6, and monocyte chemo-attractant protein 1 (MCP 1). ^15^ In contrast, deletion of the *FABP4* gene protects against the inflammatory activity of macrophages and adipocytes. Furthermore, inhibition of FABP4 through pharmacological intervention was found to mitigate LPS-induced tissue damage and improve the survival rate in mice. ^16^ Previous studies have shown that systemic infection jeopardizes the liver by damaging parenchymal cells. In response to the FABP4-induced release of proinflammatory cytokines, especially TNF-α, Kupffer cells in the liver produce IL-6. ^17^^,^^18^

The pathophysiology of sepsis can be studied in a sepsis mouse model injected with bacterial lipopolysaccharide (LPS). ^19^ LPS is the main endotoxin component of the membrane of gram-negative bacteria. It activates macrophages by triggering the toll-like receptor 4 (TLR4) signaling pathway within Kupffer cells and inducing inflammatory cytokine release. ^20^ LPS play a vital role in acute and chronic inflammation, ^21^ including that caused by gram-negative bacteria in sepsis. ^19^

*Imperata cylindrica* (L.), commonly known as cogon grass, is used to treat multiple conditions, such as fever, hepatitis, dysentery, diarrhea, hepatitis, typhus muscle pain, cancer, and hypertension. ^22^^–^^24^ Phytochemical screening has shown that extract of cogon grass root (ECGR) contains potent antioxidants, including isoeugenin, tannins, saponins, flavonoids, terpenoids, and alkaloids. ^24^^,^^25^ Specifically, isoeugenin demonstrates potent antioxidant activity through nitrite oxide (NO) scavenging, significantly inhibiting the expression of inducible nitric oxide synthase (iNOS), cyclooxygenase-2 (COX-2), and proinflammatory mRNA, which play essential roles in sepsis. ^26^ However, the use of cogon grass root for the treatment of sepsis has not been explored. Therefore, this study aimed to investigate the effect of ECGR on a mouse model of sepsis. We hypothesized that ECGR would ameliorate sepsis by reducing inflammatory responses and oxidative damage via antioxidant activity.

## Methods

### Ethical consideration

Ethical clearance was granted by the Research Ethics Committee of Universitas Padjadjaran, Bandung, Indonesia (approval number: 921/UN6.KEP/EC/2019).

### ECGR

Cogon grass was obtained from Solo, Central Java, Indonesia, and its authenticity was tested by the Bandung Institute of Technology. The roots of the cogon grass were separated, washed with water, and dried for two weeks. The roots were then macerated, filtered, and extracted. The extract was filtered and separated from the solvent using a vacuum rotary evaporator R220 pro (BUCHI Indonesia, Tangerang, Indonesia). The ECGR was then diluted in 0.5% carboxymethylcellulose (CMC) (Merck, U.S.A) and administered at a dose of either 90 or 115 mg/kg body weight (BW), as in our previous study. ^27^

### Model and research design

Male mice ( *Mus musculus sp.*) of the *Deutsche Denken Yoken* strain (8–10 weeks of age, 30–35 g body weight) were provided by the Biofarma Company (Bandung, Indonesia). All efforts were made to relieve any pain and distress of the animals by strictly following the procedures. The mice were acclimatized for seven days in the laboratory. They were then kept in cages at the animal laboratory of Universitas Padjadjaran at a controlled room temperature and on a 12 hours light/12 hours dark cycle. The mice were provided regular food, drinking water ad libitum, observed daily to confirm lack of behavior, and weighed every three days. This study was conducted following the ARRIVE Essential 2.0 checklist for pre-clinical animal studies.

A randomized post-test control group design was used. Determination of the number of samples for each treatment group was determined using the Frederer formula. The mice were divided into the following four experimental groups (5–8 mice per group; 29 mice total): group 1 (control), mice treated with CMC 0.5% (the solvent of ECGR); group 2, mice treated with CMC 0.5% + 10 μL/kg BW LPS; group 3, mice treated with 10 μL/kg BW LPS + 90 mg/kg BW ECGR; and group 4, mice treated with 10 μL/kg BW LPS + 115 mg/kg BW ECGR.

Briefly, groups 1 and 2 were treated with 0.5% CMC, while groups 3 and 4 were treated with ECGR in 0.5% CMC at doses of 90 and 115 mg/kg BW, respectively, for two weeks. The mice were weighed every 3 days to determine the effects of ECGR on body weight. The ECGR solutions were administered daily for two weeks, between 3 and 5 pm. ^27^ After two weeks, groups 2, 3, and 4 were injected intraperitoneally with LPS (10 μL//kg BW; Sigma-Aldrich, St. Louis, MO) diluted in 50 μL PBS, as in previous studies. ^28^ At 8 hours after injection, ^29^ the mice were euthanized by cervical dislocation, and portions of their livers, spleens, and hearts were snap-frozen in liquid nitrogen and stored at −80 °C until further use. The remaining liver, spleen, and heart tissues were processed for histological examination.

### Measurement of blood parameters

Blood was collected from the inferior vena cava. A complete blood count profile (CBCP) was drawn automatically using the CLIA Waived Hematology Analyzer Sysmex XW-100 (Sysmex America, Lincolnshire, U.S.A). Serum was separated by centrifugation at 1,500 × *g* for 15 minutes at 4 °C, and the lysates were stored at −80 °C until further examination.

### Histological analysis of the livers, hearts and spleen

Liver, spleen, and heart tissues were prepared for histopathological evaluation using the Kiernan method. ^30^ Briefly, they were fixed in 4% paraformaldehyde, embedded in paraffin, and stained with hematoxylin and eosin (H&E) according to the Harris method. ^31^

### GPx3 activity assay

GPx3 activity was measured using a commercial kit Randox-Backpack RS 505 (RANDOX Laboratories Ltd, Antrim, U.K), following the method described by Paglia and Valentine (1967). ^32^ Briefly, Glutathione Peroxidase (GPx) catalyses the oxidation of Glutathione (GSH) by Cumene Hydroperoxide. In the presence of Glutathione Reductase (GR) and NADPH the oxidised Glutathione (GSSG) is immediately converted to the reduced form with a concomitant oxidation of NADPH to NADP+. The decrease in absorbance at 340 nm is measured.

### FABP4 measurement

FABP4 levels in the samples were measured using commercial enzyme-linked immunosorbent assay (ELISA) kits Mouse FABP4 ELISA Kit, catalog number EM1506 (Fine Biotech, Wuhan, China) according to the manufacturer's protocol.

### RNA isolation and reverse transcription (RT)–PCR

Total RNA was isolated from the organs using TRIzol™ Reagent catalog number 15596026 (Invitrogen, Massachusetts, US). DNA synthesis was performed using ReverTra AceTM qPCR RT Master Mix with gDNA Remover product number FSQ-301 (Toyobo, Osaka, Japan) according to the manufacturer's protocol. Quantitative real-time RT-PCR was performed using the SensiFAST™ SYBR ^®^ No-ROX Kit product number BIO-98020 (Bioline, United Kingdom) according to the manufacturer's instructions and used Rotor-Gene Q quantitative real-time PCR machine (Qiagen, USA). The first step of Quantitative real-time RT-PCR was polymerase activation for one cycle at 95 °C for two minutes, then denaturation prosses at 95 °C for five seconds and annealing at 60 °C for 30 seconds. The denaturation and annealing process took 40 cycles. The PCR was performed using mGAPDH as a housekeeping gene and the primers from Integrated DNA Technologies, USA. The gene-specific primers for the cDNA used in this study are listed in Table 1.

**Table 1.** Primers for quantitative real-time PCR.

Gen	Forward	Reverse
mIL-1β	5′-AACCTGCTGGTGTGTGACGTTC	5′-CAGCACGAGCTTTTTTGTTGT
mIL-10	5′-ATGCAGGACTTTAAGGGTTACTTGGGT-3′	5′-ATTTCGGAGAGAGGTACAAACGAGGTTT-3′
mIL6	5′-CCTCTGGTCTTCTGGAGTACC-3′	5′-ACTCCTTCTGTGACTCCAGC-3′
Mgapdh	AGCCCCCAGTCTGTATCCTT	TCCACCACCCTGTTGCTGTA
mSOD1	5′-TGC GTG CTG AAG GGC GAC-3′	5′-GTC CTG ACA ACA CAA CCT GGT TC-3
MSOD2	5′-GGA GCA AGG TCG CTT ACA GA-3′	5′-GTG CTC CCA CAC GTC AAT C-3′
mTNF-α	5′-TGAGGTCAATCTGCCCAAGT-3′	5′-CTGAGCCATAATCCCCTTTCTA-3′

### Statistical analysis

Statistical analyses were performed using the GraphPad Prism version 7.0a for Mac (GraphPad Software, Inc. CA, 92037 USA). Normally distributed data are represented as the mean ± SD and skewed data as the median ± interquartile range (IQR). Normality was assessed using the Shapiro-Wilk test. The p-values were calculated using analysis of variance (ANOVA) with Tukey’s post hoc test for normal distributions and the Kruskal-Wallis test with Dunn’s post hoc test for skewed distributions. Differences were considered statistically significant at p < 0.05 (for two-tailed p-values).

## Results

**Figure 1.**
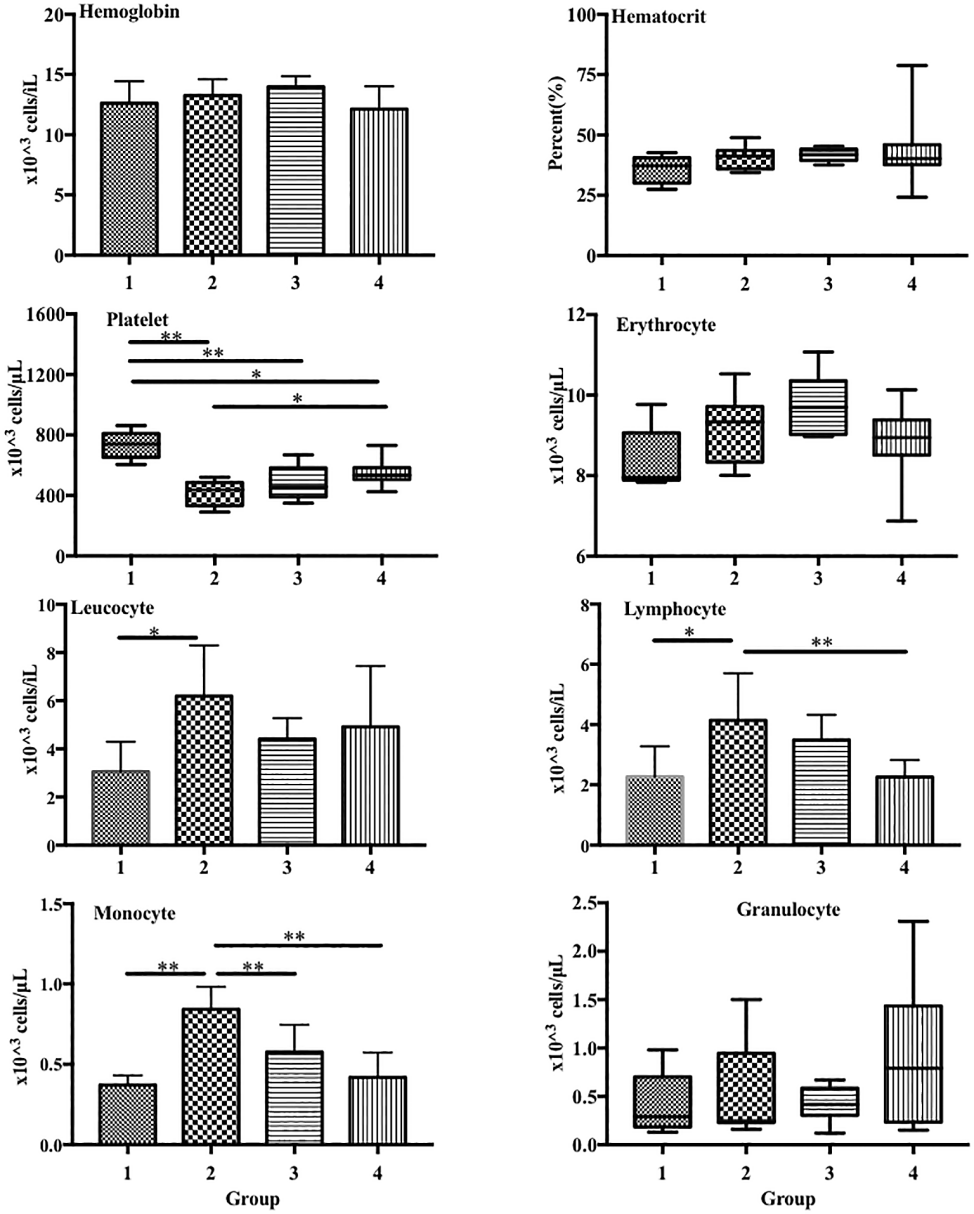
ECGR pre-treatment increased platelet levels and decreased lymphocyte and monocyte levels in a sepsis mouse model. Group 1, control; group 2, mice stimulated with LPS; groups 3 and 4, mice treated with 90 and 115 mg/kg BW ECGR, respectively, and stimulated with LPS. Data are represented as the mean ± SD, n = 5–8 per group. One-way ANOVA with Tukey's post hoc test and the Kruskal-Wallis test with Dunn's post hoc test were performed for normal and skewed data, respectively. *p < 0.05, significant; **p < 0.01, very significant.

In the blood profiles, platelet levels were significantly lower in group 2 (LPS treatment only) than in group 1 (control) (417.6 × 10 ^3^ vs. 732.60 × 10 ^3^ cells/μL, p < 0.01). Additionally, platelet levels were significantly higher in group 4 (115 mg/kg ECGR) than in group 2 (550.3 × 10 ^3^ vs 417.6 × 10 ^3^ cells/μL, p < 0.05; Figure 1). Conversely, LPS induced a substantial increase in the lymphocyte and monocyte counts in group 2 compared with those in the control (lymphocytes: 4.13 × 10 ^3^ vs. 2.26 × 10 ^3^ cells/μL, p < 0.05; monocytes: 0.84 × 10 ^3^ vs. 0.37 × 10 ^3^ cells/μL, p < 0.05). In addition, a significant decrease in the lymphocyte count was observed in group 4 compared with that in group 2 (2.26 × 10 ^3^ vs. 4.13 × 10 ^3^ cells/μL, p < 0.05). Furthermore, significant decreases in the monocyte count were observed in groups 3 and 4 compared with that in group 2 (0.43 × 10 ^3^ and 0.29 × 10 ^3^, respectively, vs. 0.84 × 10 ^3^ cells/μL, p < 0.05). Finally, a significant increase in the leukocyte count was observed in group 2 compared with that in group 1 (6.19 × 10 ^3^ vs. 3.05 × 10 ^3^ cells/μL, p < 0.05). However, no significant difference in the leukocyte count was observed between the treatment groups ( Figure 1).

**Figure 2.**
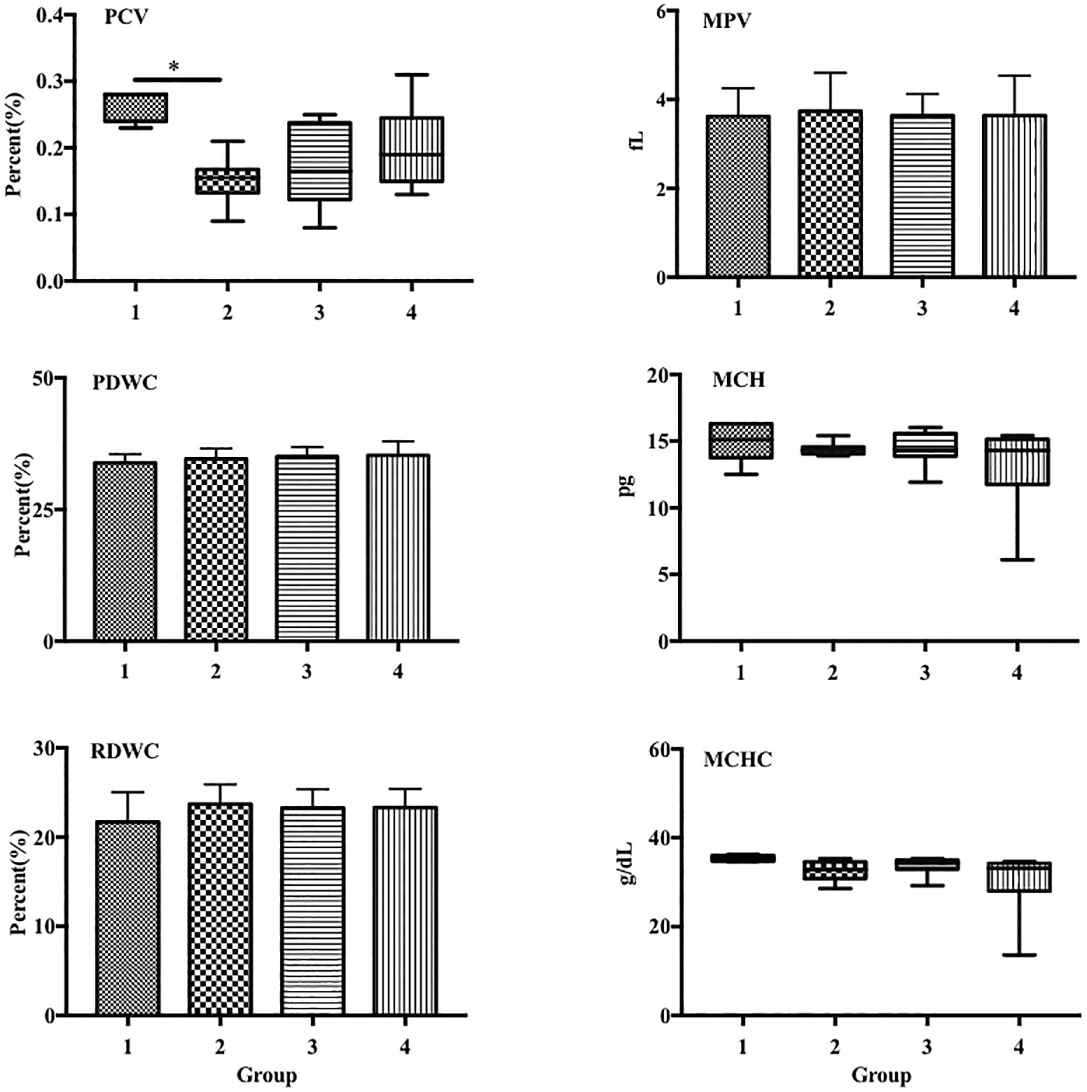
LPS decreased PCV in a sepsis mouse model. Group 1, control; group 2, mice stimulated with LPS; groups 3 and 4, mice treated with 90 and 115 mg/kg BW ECGR, respectively, and stimulated with LPS. Data are represented as the mean ± SD, n = 5–8 per group. One-way ANOVA with Tukey's post hoc test and the Kruskal-Wallis test with Dunn's post hoc test were performed for normal and skewed data, respectively. *p < 0.05, significant; **p < 0.01, very significant. Abbreviations: PCV, packed cell volume; MPV, mean platelet volume; PDW, platelet distribution width; MCV, mean corpuscular volume; MCH, mean corpuscular hemoglobin; MCHC, mean corpuscular hemoglobin concentration.

Group 2 exhibited a significantly decreased percentage of packed cell volume (PCV) compared with that in the control (0.15% vs. 0.26%, Figure 2). Additionally, group 4 also showed a decreased PCV percentage compared with that in group 2, although the difference was not statistically significant.

**Figure 3.**
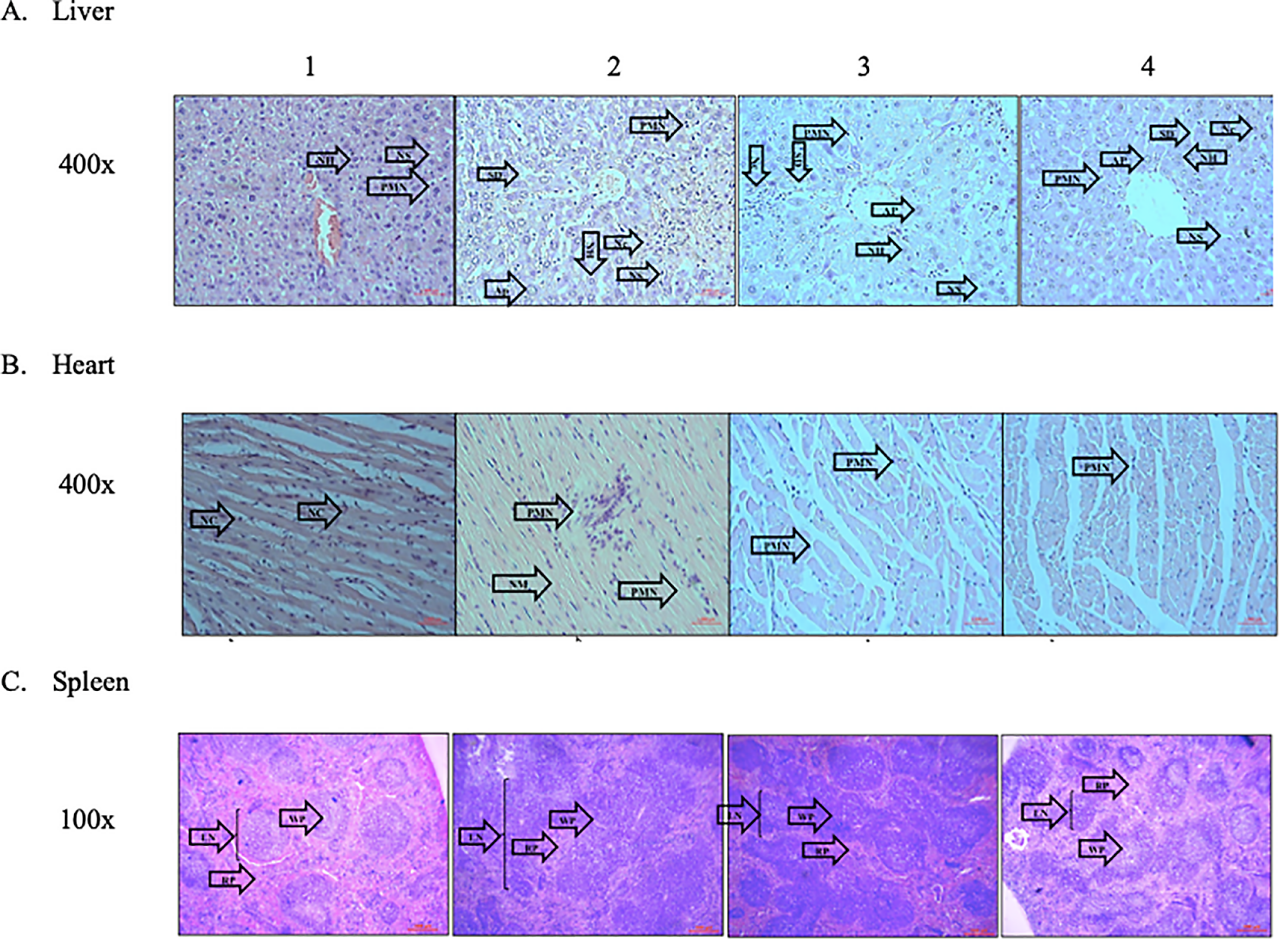
Effect of ECGR on the histological characteristics of the liver, heart, and spleen of a sepsis mouse model. Group 1, control; group 2, mice stimulated with LPS; groups 3 and 4, mice treated with 90 and 115 mg/kg BW ECGR, respectively, and stimulated with LPS. Abbreviations: Nc, necrotic area of hepatocyte; Ap, apoptotic hepatocytes; SD, sinusoidal dilatation; PMN, polymorphonuclear cell; NS, normal sinusoid; NH, normal hepatocyte; NM, necrotic area of the myocardium; VD, vascular dilatation; NC, normal cardiomyocyte; LN, lymph node; WP, white pulp; RP, red pulp.

The typical architectural structures of the liver, heart, and spleen tissues were observed in group 1. Additionally, neither necrosis or apoptosis were observed, and the hepatic sinusoids and heart vessels were not dilated. In group 2, abnormalities in the architectural structures of the three organs were observed. Several areas of necrosis, cellular swelling, vessel dilation, and inflammatory cell infiltration, especially that of PMNs, were observed. In contrast, fewer areas of necrosis and cellular swelling were observed in the treatment groups (groups 3 and 4). In addition, the treatment groups demonstrated reduced PMN infiltration ( Figure 3).

**Figure 4.**
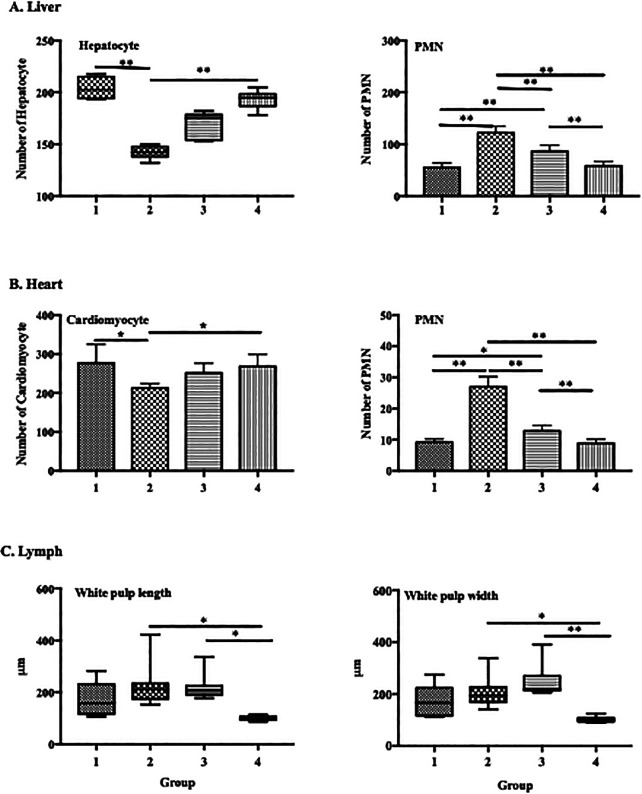
Effect of ECGR on the histological quantification of the liver, heart, and spleen of a sepsis mouse model. Group 1, control; group 2, mice stimulated with LPS; groups 3 and 4, mice treated with 90 and 115 mg/kg BW ECGR, respectively, and stimulated with LPS. Data are represented as the mean ± SD, n = 5–8 per group. One-way ANOVA with Tukey's post hoc test and the Kruskal-Wallis test with Dunn's post hoc test were used for normal and skewed data, respectively. *p < 0.05, significant; **p < 0.01, very significant; ***p < 0.001, extremely significant.

ECGR significantly affected the numbers of PMN cells and hepatocytes in the liver tissues. In the mice treated with only LPS (group 2), an increase in the number of PMN cells and significant decrease in that of hepatocytes were observed. Conversely, a decrease in the number of PMN cells and significant increase in that of hepatocytes were observed in the groups treated with ECGR (groups 3 and 4; Figure 4).

Similar conditions were observed in the heart, with significant differences in cardiomyocyte numbers in groups 1 and 4 compared with those in group 2 (277.3 and 251.6 vs. 212.5 cells; p < 0.01). There was also increased dilatation in the length and width of the white pulp spleen vasculature in the group treated with LPS compared with that in the control group, although the difference was not statistically significant. In contrast, there was a significant difference in length (p < 0.05) and width (p < 0.01) between groups 2 and 4 ( Figure 4).

**Figure 5.**
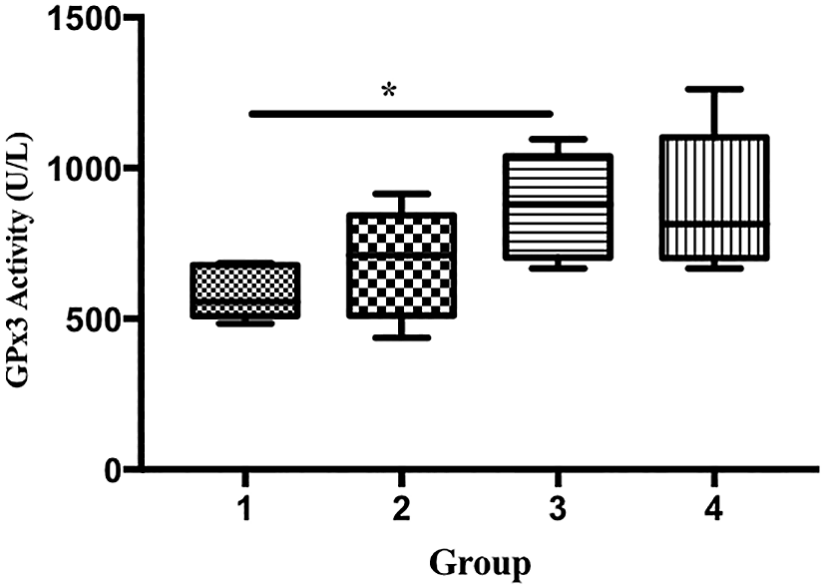
Effect of ECGR on GPx3 activity. Group 1, control; group 2, mice stimulated with LPS; groups 3 and 4, mice treated with 90 and 115 mg/kg BW ECGR, respectively, and stimulated with LPS. Data were analyzed via the Kruskal-Wallis test with Dunn's post hoc test. *p < 0.05, significant.

**Figure 6.**
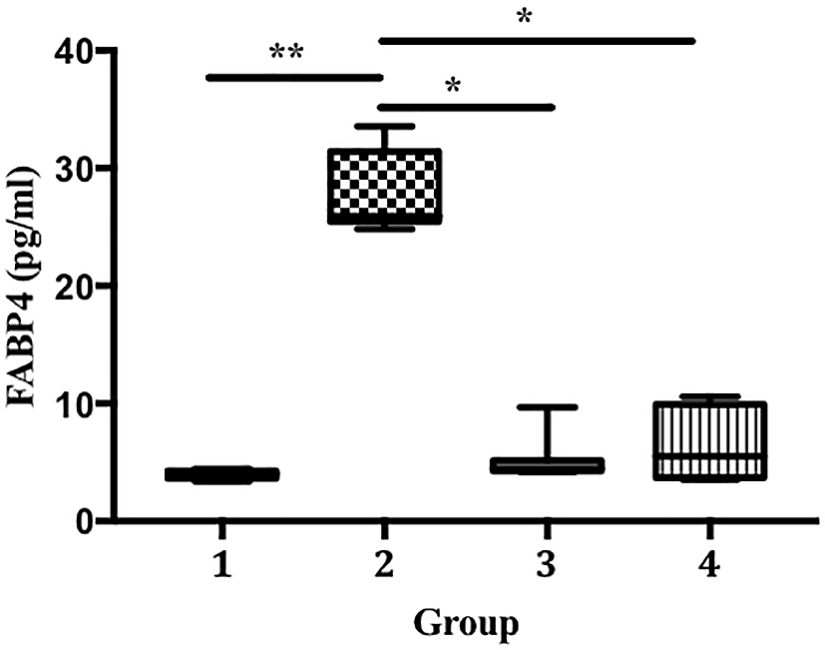
Suppression of FABP4 expression in an LPS-induced sepsis mouse model pre-treated with ECGR. Group 1, control; group 2, mice stimulated with LPS; groups 3 and 4, mice treated with 90 and 115 mg/kg BW ECGR, respectively, and stimulated with LPS. Data were analyzed via the Kruskal-Wallis test with Dunn's post hoc test. *p < 0.05, significant.

The GPx activity of the mice in group 3 was significantly higher than that in group 1 (585.9 vs. 876.5 U/L), p < 0.05; Figure 5). We also found that the LPS-treated groups developed significantly higher FABP4 expression than the control (27.69 vs. 3.98 pg/mL, p < 0.01; Figure 6). Compared with that in group 2, FABP4 expression was suppressed in groups 3 and 4 (the ECGR treatment groups) (27.69 vs. 5.19 and 6.55 pg/mL, p < 0.05).

**Figure 7.**
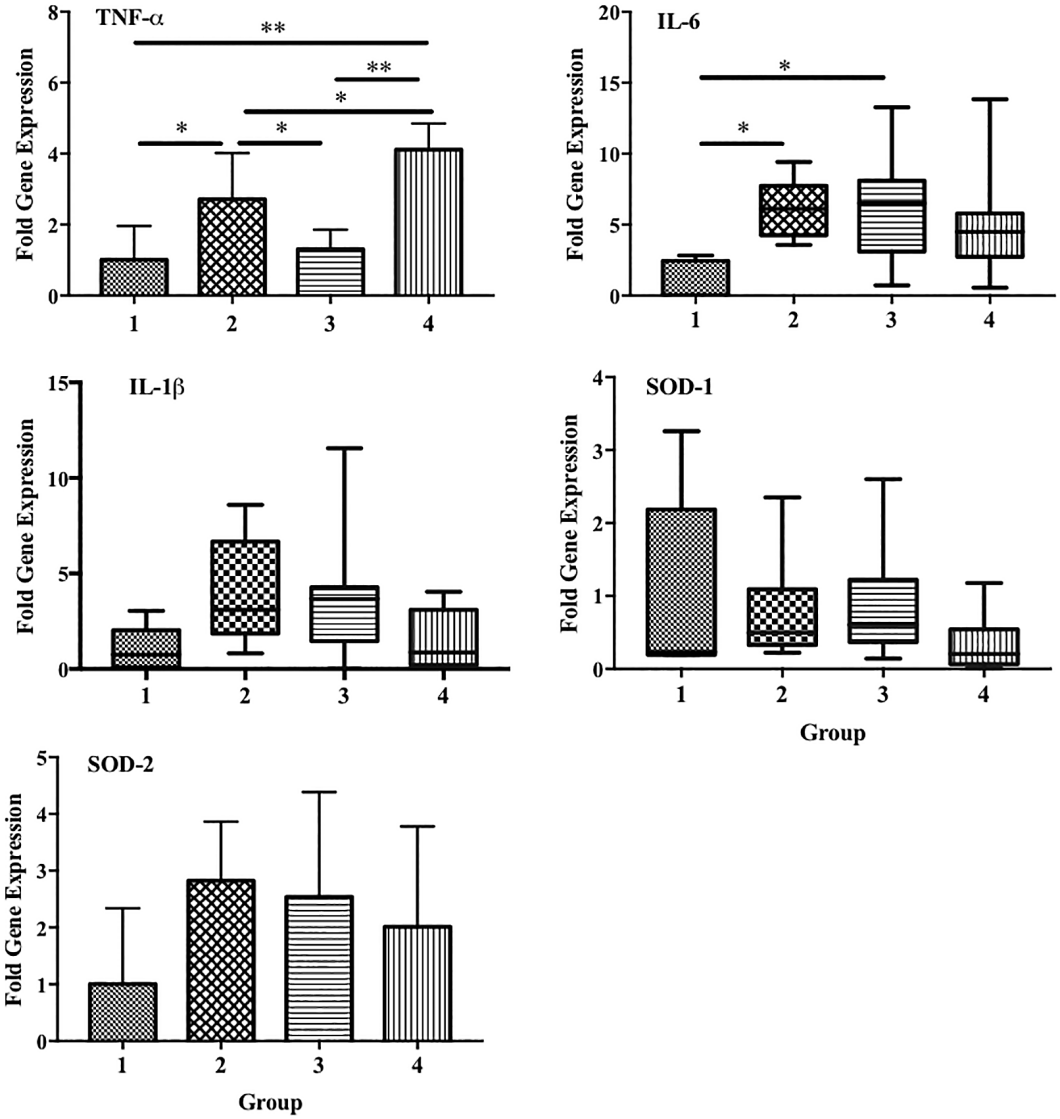
Effect of ECGR on the expression of several genes associated with inflammation and antioxidant properties. Group 1, control; group 2, mice stimulated with LPS; groups 3 and 4, mice treated with 90 and 115 mg/kg BW of ECGR, respectively, and stimulated with LPS. Data are represented as the mean ± SD, n = 5–8 per group. One-way ANOVA with Tukey's post hoc test and the Kruskal-Wallis test with Dunn's post hoc test were performed for normal and skewed data, respectively. *p < 0.05, significant; **p < 0.01, very significant.

We also observed a significant increase in the expression of TNF-α and IL-6 in group 2 compared with that in the control group (p < 0.05). However, a significant decrease in TNF-α expression was observed in group 3 compared with that in group 2 (p < 0.05). Decreased expression of IL-6 and IL-1β was also observed in group 4, although the differences were not statistically significant. No significant differences in the expression of SOD1 and SOD2 were observed among the groups ( Figure 7).

## Discussion

Overall, ECGR treatment provoked robust increases in platelet levels, GPx3 activity, hepatocyte numbers, and cardiomyocyte numbers. Treatment also induced decreases in lymphocyte numbers, monocyte numbers, TNF-α levels, and FABP4 levels. Furthermore, the expression of other related septic proinflammatory cytokines, including IL-6 and IL-1β, was also decreased by ECGR.

Sepsis is a systemic response that endangers the body, causing organ hypofunction and even death. ^33^ The pathogenesis of sepsis is complex due to the involvement of various immune components. In experimental animals, LPS treatment causes the same pathophysiological changes as sepsis in humans. ^34^ This is due to the presence of LPS-binding protein (LBP) in the blood and extracellular fluid, which binds to lipid A (the bioactive part of LPS) and carries LPS to cluster of differentiation 14 (CD14) in monocytes, macrophages, and neutrophils. ^35^ The interaction between the LBP-LPS complex and the CD14 receptor allows LPS to bind to TLR4, signaling the cell nucleus to stimulate the production and release of inflammatory mediators. ^36^ These inflammatory mediators activate the endothelium, causing increased expression of adhesion molecules, such as selectin-E, intracellular adhesion molecule-1 (ICAM-1), and vascular cell adhesion molecule-1 (VCAM-1), which serve as ligands for leukocyte integrins. The proinflammatory cytokines also enhance the proliferation and differentiation of naive T lymphocytes into effector T lymphocytes. In addition, TNF-α and IL-1 increase the secretion of chemokines, such as CXCL1 and CCL2, which bind to neutrophils and monocytes, respectively. This increases the affinity of leukocyte integrins for their ligands and enhances leukocyte migration. ^36^^,^^37^

In this study, leukocyte, monocyte, and lymphocyte counts were observed to be significantly increased in the group treated with LPS alone (group 2) compared with those in the control (group 1; Figure 1). However, no significant differences were observed between these cell counts in the treatment groups (groups 3 and 4) and those in group 2. Increased PMN cell counts were also observed in group 2 via histopathological quantification ( Figures 3, 4). In addition, significant decreases in the lymphocyte and monocyte counts were observed in the treatment groups compared with those in group 2 ( Figure 1). The PMN cell count in groups 3 and 4 was also decreased compared with that in group 2, as observed in the histopathological analysis ( Figure 4). These observations were likely due to ECGR treatment.

Previous studies have found that ECGR contains phenols in the form of flavonoids and isoeugenin. ^25^^,^^26^^,^^38^ Phytochemical screening has shown that the flavonoid content of cogon grass root is 4.8%. ^38^ Hyo-Jin An *et al*. also reported the isoeugenin content of cogon grass as approximately 0.268 mg/g dry ECGR. ^26^

Flavonoids are known to inhibit inflammatory reactions. Their mechanism may involve the recruitment and regulation of neutrophils through chemokines, IL-8, and leukotriene B4. ^39^ This mechanism has also been observed in isoeugenol, which specifically decreases the expression of proinflammatory cytokines. ^26^ This is consistent with our results, which demonstrated significant decreases in TNF-α expression in group 3. IL-6 and IL-1β expression was also decreased in group 4, although not significantly ( Figure 7). Thus, ECGR demonstrated potential anti-inflammatory activity through the inhibition of lymphocytes, monocytes, and PMN cell infiltration in the livers and hearts of mice with LPS-induced sepsis.

Proinflammatory cytokines play essential roles in sepsis through several pathways. Specifically, activation of the TLR4 signaling pathway ^21^ causes plasminogen stimulation and the activation of antithrombin III in the fibrinolysis system. These effects trigger fibrinolysis, causing depletion of fibrinogen substances, induction of disseminated intravascular coagulation, and increased platelet damage. ^40^ The pathogenesis of sepsis also involves the formation of free radicals. Endotoxins produced during sepsis induce mitochondrial production of reactive oxygen species (ROS), including superoxide, hydrogen peroxide, and hydroxyl. ^34^^,^^41^ This further stimulates ROS production in endothelial cells, leading to perpetual free radical production. ROS cause macrostructural changes in the mitochondria that ultimately lead to the dysfunction of multiple organs. ^41^ In addition, ROS production induces damage through the pathological redox cycle, which occurs independently, leading to cell damage and enhanced apoptosis of cardiomyocytes and hepatocytes. ^42^^,^^43^

The flavonoids and isoeugenin in ECGR also demonstrate antioxidant properties through the inhibition of NO activity and iNOS, COX-2, and nuclear factor-kappa B (NF-κB) expression. These substances play essential roles in the perpetuation of the pathological redox cycle that damages cells. ^39^^,^^44^ Thus, the sustained numbers of hepatocytes and cardiomyocytes in the sepsis mouse model may be attributed to the two-week ECGR pre-treatment. ^45^ In this study, group 4 demonstrated improvement in the liver and heart tissues and increased hepatocyte and cardiomyocyte numbers compared with those in group 2 ( Figures 3, 4).

Flavonoid antioxidants are considered secondary, exogenous, or non-enzymatic antioxidants. Primary antioxidants, such as SOD, catalase (Cat), and GPx, are produced endogenously. Deficiencies in GPx, which is the first-line defense against oxidative stress, have previously been found to be associated with sepsis. ^46^ In this study, the GPx3 activity in group 3 was significantly higher than that in group 1 ( Figure 5). In contrast, no significant differences in the expression of SOD1 and SOD2 were observed among the groups ( Figure 7).

Enhanced proinflammatory release and LPS activity in sepsis have been found to cause immunologic and metabolic alterations, especially of lipid metabolism. ^47^^,^^48^ Higher fatty acid levels in patients with sepsis are associated with increased lipolysis due to reductions in mitochondrial acyl-CoA synthetase (ACS), which aids the synthesis of triglycerides for energy storage. LPS and cytokine release are also associated with lower ACS expression, thereby assisting the mobilization of FAs. ^12^ A previous study has shown that increased levels of FABP4 are associated with a robust inflammatory response in septic conditions. ^15^ We observed a remarkable increase in FABP4 levels after LPS induction in group 2. In contrast, the mice pre-treated with ECGR (groups 3 and 4) exhibited decreased FABP4 expression ( Figure 6). This may be attributed to the ability of FABP4 to modulate the NF-kB pathway, resulting in the expression of inflammasome complexes (e.g., NLR family pyrin domain containing 3 [NLRP3] and pro-IL-1β). In addition, NLRP3 activation is associated with the overproduction of IL-1β, resulting in detrimental effects in sepsis. ^49^^,^^50^ Inhibition of FABP4 expression through surgical and pharmacological interventions may alleviate LPS-induced tissue damage. ^15^^,^^51^

Cogon grass root contains flavonoids that phenotypically demonstrate anti-inflammatory effects. The previous study was measured total flavonoids as quercetin in cogon grass; the value was 7.35 QE/g dry wt. ^52^ One of the flavonoid derivatives in the cogon grass is quercetin, which presented 36.47 mg/100 DW. ^53^ Quercetin and icariin, which are kaempferol derivatives, have been shown to demonstrate prominent inhibition of FABP4 expression. ^54^^,^^55^ The attenuation of FABP4 in macrophages subsequently inhibits the NF-kB pathway through induction of SIRT3 and diminishes proinflammatory cytokine production. ^51^^,^^56^ Quercetin also suppresses NF-kB activation and blocks NLRP3 inflammasome activation. ^57^ These findings suggest that cogon grass root may exhibit direct and indirect inhibitory effects on FABP4 and associated protein complex expression in inflammatory pathways. The limitation of this study was that we did not measure the active substance of ECGR, downstream protein products of our target genes and did not pursue an in vitro study to explore the mechanism of ECGR in sepsis conditions.

## Conclusions

In this study, we established that ECGR played a beneficial role in mitigating severe conditions in a sepsis mouse model. This suggests that cogon grass root may ameliorate sepsis by increasing the platelet level, GPx3 activity, hepatocyte count, and cardiomyocyte count, as well as by reducing the lymphocyte count, monocyte count, TNF-α expression, and FABP4 level. Further understanding of the mechanisms involved in the metabolic and inflammatory effects of cogon grass root is needed. This requires continued exploration of the TLR pathway and other metabolic markers associated with sepsis. Our findings emphasized the potential of cogon grass root as an herbal medicine for sepsis.

## Data availability

### Underlying data

Figshare: Experimental flow chart: https://doi.org/10.6084/m9.figshare.23897076. ^58^

Figshare: Data measurement of Sepsis Mice Model with Ethanol Extract of Cogon Grass Root ( *Imperata cylindrica L.*) pre-treament, https://doi.org/10.6084/m9.figshare.16530654.v8. ^59^

Figshare: Original unedited histopathology image of the liver, heart, and spleen of a sepsis mouse model after pre-treatment with ECGR, https://doi.org/10.6084/m9.figshare.16894813.v2. ^60^

This project contains the following underlying data:

- Spleen, group 4, 115 mgkg BW ECGR + LPS.jpg
- Spleen, group 3, 90 mgkg BW ECGR + LPS.jpg
- Spleen, group 2, mice stimulated with LPS.jpg
- Spleen, group 1, control.jpg
- Heart, group 4, 115 mgkg BW ECGR + LPS.jpg
- Heart, group 3, 90 mgkg BW ECGR + LPS.jpg
- Heart, group 2, mice stimulated with LPS.jpg
- Heart, group 1, control.jpg
- Liver, groups 4, 115 mgkg BW ECGR + LPS.jpeg
- Liver, groups 3, 90 mgkg BW ECGR + LPS.jpeg
- Liver, group 2, mice stimulated with LPS.jpeg
- Liver, group 1, control.jpeg

### Reporting guidelines

Figshare: ARRIVE Essential 10 checklist-Effect of cogon grass root ethanol extract on fatty acid binding protein 4 and oxidative stress markers in a sepsis mouse model, https://doi.org/10.6084/m9.figshare.16895506.v2. ^61^

Data are available under the terms of the Creative Commons Zero “No rights reserved” data waiver (CC0 1.0 Public domain dedication).
