# Supplementary material for: Effect of cogon grass root ethanol extract on fatty acid binding protein 4 and oxidative stress markers in a sepsis mouse model
Source: F1000Res. 2023 Oct 3;10:1161. Originally published 2021 Nov 16. [Version 3] doi: 10.12688/f1000research.73561.3 (PMC10980860; doi:10.12688/f1000research.73561.3)
Supplement: Peer review history

# Reviewer report: Reviewer response for version 3

**DOI:** 10.5256/f1000research.155211.r255954

**Published:** 2024-03-29

**Authors:** Patipark Kueanjinda

**Affiliations:** 1 Chulalongkorn University, Bangkok, Thailand

**Summary**

 This study investigates the potential effects of cogon grass root ethanol extract (ECGR) on fatty acid-binding protein 4 (FABP4) and oxidative stress markers in a sepsis mouse model. The authors studied the effects of ECGR in four mouse groups: control, LPS, LPS + low-dose ECGR, and LPS + high-dose ECGR. The inflammatory cytokines, TNFalpha and IL-6, and FABP4 decreased significantly after ECGR treatment. While the study has merit, several points require clarification and refinement.

**Major comments:**

 1. The significant increase in TNFα levels after a high dose of ECGR treatment should be discussed.

 2. Please consider including a diagram illustrating how ECGR might hypothetically inhibit inflammation based on the literature review. The authors may suggest potential ECGR components that could target FABP4 by comparing their molecular structures to known FABP4 inhibitors found in the literature. This will strengthen the connection between the findings and the title.

**Minor comments:**

 1. Include the chemical profile of ECGR (using techniques like TLC or HPLC) to establish its authenticity.

 2. Specify the ECGR administration method (e.g., intragastric) and the type of LPS used (e.g., E. coli, Salmonella)

 3. Provide a clear description of the quantitative measurement methods used for the histopathological images.

 4. In Table 1, please correct the typo in the first column header ("Gen" -> "Gene") and ensure consistent formatting of primer names and sequences (i.e., -3').

 5. Please describe the specific procedures and essential reagents for each test, regardless of references to kits or previous studies. This enhances reproducibility.

 6. Figure 2, please double-check the interpretation of Group 4's PCV percentage in comparison to Group 2.

 7. Figure 7, please clarify the comparison group for Group 4 when discussing decreased cytokine expression.

 8. Figure 3, please elaborate on observed "typical architectural structures" in Group 1, possibly by specifying size, shape, form, or features of cell bodies and nuclei. Clarify quantification methods and specify if images are representative of replicates. Explain why observable necrotic or apoptotic cells were not quantified.

 9. In discussion, rephrase the second statement from "Treatment also induced decreases in lymphocytes..." to "Treatment decreased lymphocytes..." for clarity. 

 10. Please use the terms "statistically significant" or "not statistically significant" instead of "very significant."

 11. Add labels (e.g., A, B, C) to subfigures in Figures 1, 2, and 7 for clarity.

 12. If possible, provide higher-resolution images. Consider using colored markers and a legend to indicate cell types instead of text within arrows. Include scale bars with their lengths in both images and legends.

Is the work clearly and accurately presented and does it cite the current literature?

Yes

If applicable, is the statistical analysis and its interpretation appropriate?

Yes

Are all the source data underlying the results available to ensure full reproducibility?

Partly

Is the study design appropriate and is the work technically sound?

Yes

Are the conclusions drawn adequately supported by the results?

Partly

Are sufficient details of methods and analysis provided to allow replication by others?

Partly

Reviewer Expertise:

Molecular Immunology

I confirm that I have read this submission and believe that I have an appropriate level of expertise to confirm that it is of an acceptable scientific standard, however I have significant reservations, as outlined above.

# Reviewer report: Reviewer response for version 2

**DOI:** 10.5256/f1000research.119900.r181154

**Published:** 2023-07-17

**Authors:** Ming Zhong

**Affiliations:** 1 Fudan University, Shanghai, Shanghai, China

I'm glad to read about the author's interesting experimental research, and I have the following questions that I hope the author can further explain:

1. Why did you choose the Deutsche Denken Yoken strain of mice?
2. Why did you select the concentrations of 90 and 115 for ECGR, and why was the treatment conducted between 3 PM and 5 PM? The cited references seem to be related to the treatment of hyperlipidemia; please provide further explanation.
3. The model seems to involve preprocessing. It is suggested to use an experimental flowchart to present the process to avoid unnecessary misunderstandings.
4. After a 2-week preprocessing period, the mice's livers and other organs were collected after an 8-hour LPS treatment. Please further explain the reasoning behind choosing this specific timing.
5. As the author mentioned preprocessing, why did you select several antioxidant genes for monitoring in Figure 7, instead of directly using Western blot to detect the downstream protein products of these genes?

Is the work clearly and accurately presented and does it cite the current literature?

Partly

If applicable, is the statistical analysis and its interpretation appropriate?

Yes

Are all the source data underlying the results available to ensure full reproducibility?

Partly

Is the study design appropriate and is the work technically sound?

Partly

Are the conclusions drawn adequately supported by the results?

Partly

Are sufficient details of methods and analysis provided to allow replication by others?

Partly

Reviewer Expertise:

ARDS and sepsis

I confirm that I have read this submission and believe that I have an appropriate level of expertise to confirm that it is of an acceptable scientific standard, however I have significant reservations, as outlined above.

# Response

**Published:** 2023-08-27

**Authors:** Mirasari Putri

**Affiliations:** Universitas Islam Bandung, Indonesia

First, thank you for the review of this article, we very much appreciate your effort. These are the answers to your questions, Also we revised the article based on your suggestions.

1. We chose the Deutsche Denken Yoken strain of mice for our study because this strain has been widely used in trials for drug efficacy and various research fields, such as pharmacological, pharmacokinetic, and toxicological studies. This is the reason why this strain is usually used as a general-purpose mouse for various studies. We have no specific purpose for choosing this strain.
2. Before we carried out this study, we had conducted several preliminary studies to select the optimal concentration, and as a result, we selected concentrations of 90 and 115 for ECGR. Why is the treatment done between 15.00 and 17.00? According to previous studies, treatment with ECGR did not have a time-dependent effect; there is no specific time. ^1^ We chose this time to expedite the technical research process.
3. Thank you for the suggestion. We have added an experimental flowchart to our revised manuscript (line 330).
4. LPS is known to be used to induce sepsis in many types of previous studies. ^2,3^ Before this current study, we conducted a study and showed that LPS had a prominent effect on septic scores 8 hours after administration. ^4^ For this reason, we chose that time. We have added this study as a citation in the methods section (line 132).
5. Based on previous studies ^5,6^ showed that the expression of certain genes sufficiently indicates that a given substance affects the target protein, even so, that we realize that measuring the concentration of the target protein directly will show more reliable results. Therefore, we include this as a limitation of our research (line 315).

References:

1. An HJ, Nugroho A, Song BM, Park HJ. Isoeugenin, a novel nitric oxide synthase inhibitor isolated from the rhizomes of Imperata cylindrica. Molecules. 2015;20(12):21336–45.
2. El Kamouni S, El Kebbaj R, Andreoletti P, El Ktaibi A, Rharrassi I, Essamadi A, et al. Protective effect of argan and olive oils against LPS-induced oxidative stress and inflammation in mice livers. International journal of molecular sciences. 2017;18(10):2181.
3. Lin MX, Yi YX, Fang PP, Huang SS, Pan CW, Jin LX, et al. Shikonin protects against D-Galactosamine and lipopolysaccharide-induced acute hepatic injury by inhibiting TLR4 signaling pathway. Oncotarget. 2017;8(53):91542.
4. Putri M, Anggraeni N, D RATM, Ramli GA, Kusmiati M, Andriane Y, et al. Cogongrass ( Imperata cylindrica L.) Ethanol Extract on Sepsis Mice Model Body Weight and Sepsis Score. Global Medical and Health Communication. 2020 Dec 30;8(3):206–10.
5. AbdAllah NB, Toraih EA, Al Ageeli E, Elhagrasy H, Gouda NS, Fawzy MS, et al. MYD88, NFKB1, and IL6 transcripts overexpression are associated with poor outcomes and short survival in neonatal sepsis. Sci Rep. 2021 Jun 28;11:13374.
6. Larrouyet-Sarto ML, Tamura AS, Alves VS, Santana PT, Ciarlini-Magalhães R, Rangel TP, et al. P2X7 receptor deletion attenuates oxidative stress and liver damage in sepsis. Purinergic Signal. 2020 Dec;16(4):561–72.

# Reviewer report: Reviewer response for version 2

**DOI:** 10.5256/f1000research.119900.r119738

**Published:** 2022-01-17

**Authors:** Katrin Roosita

**Affiliations:** 1 Department of Community Nutrition, Faculty of Human Ecology, IPB University, Bogor, Indonesia

The authors have addressed appropriately my concerns that were raised in the peer review report.

Is the work clearly and accurately presented and does it cite the current literature?

Yes

If applicable, is the statistical analysis and its interpretation appropriate?

Partly

Are all the source data underlying the results available to ensure full reproducibility?

Yes

Is the study design appropriate and is the work technically sound?

Yes

Are the conclusions drawn adequately supported by the results?

Partly

Are sufficient details of methods and analysis provided to allow replication by others?

Partly

Reviewer Expertise:

Nutrigenomic, Nutraceuticals, herbal medicine, stress oxidative and human nutrition.

I confirm that I have read this submission and believe that I have an appropriate level of expertise to confirm that it is of an acceptable scientific standard.

# Reviewer report: Reviewer response for version 1

**DOI:** 10.5256/f1000research.77221.r100358

**Published:** 2021-12-14

**Authors:** Katrin Roosita

**Affiliations:** 1 Department of Community Nutrition, Faculty of Human Ecology, IPB University, Bogor, Indonesia

This manuscript has an acceptable scientific standard, but I have concerns about some details, as follows:

1. How was the antioxidant activity measured/determined?
2. Please elaborate/explain the parameters of the immune system-associated biomarker levels that were also measured.
3. This study aimed to assess the effects of ECGR on FABP4 and oxidative stress-related factors, meanwhile, the conclusion only mentions that cogon grass root may aid in the development of herbal medicines and specific treatments for sepsis, not specifically addressing the purpose of this study.
4. It will also be valuable to explain the concentration of flavonoid antioxidants as bioactive compounds, such as Quercetin and icariin if authors have information about these.

Is the work clearly and accurately presented and does it cite the current literature?

Yes

If applicable, is the statistical analysis and its interpretation appropriate?

Partly

Are all the source data underlying the results available to ensure full reproducibility?

Yes

Is the study design appropriate and is the work technically sound?

Yes

Are the conclusions drawn adequately supported by the results?

Partly

Are sufficient details of methods and analysis provided to allow replication by others?

Partly

Reviewer Expertise:

Nutrigenomic, Nutraceuticals, herbal medicine, stress oxidative and human nutrition.

I confirm that I have read this submission and believe that I have an appropriate level of expertise to confirm that it is of an acceptable scientific standard, however I have significant reservations, as outlined above.

# Response

**Published:** 2021-12-30

**Authors:** Mirasari Putri

**Affiliations:** Universitas Islam Bandung, Indonesia

We greatly appreciate the reviewer’s time and effort in providing useful feedback and corrections. These are our answers:

 1. How was the antioxidant activity measured/determined?

**Response:** We measured the antioxidant by measuring the activity of Glutathione peroxidase 3 (GPx3 ) and superoxide dismutase gene expression (SOD). In mammals, GPx and SOD are the main antioxidants that protect cells from damage caused by free radicals through synergistic action. We have revised the abstract (Line 26)

 2. Please elaborate/explain the parameters of the immune system-associated biomarker levels that were also measured.

**Response:** We measured gene expression of TNF-α, IL-6, and IL-1β, we revised the abstract (Line 27)

 3. This study aimed to assess the effects of ECGR on FABP4 and oxidative stress-related factors, meanwhile, the conclusion only mentions that cogon grass root may aid in the development of herbal medicines and specific treatments for sepsis, not specifically addressing the purpose of this study.

**Response:** We have revised the conclusion according to the purpose of this study (Line 35)

 4. It will also be valuable to explain the concentration of flavonoid antioxidants as bioactive compounds, such as Quercetin and icariin if authors have information about these.

**Response:** In our study, we did not isolate the bioactive compounds. Still, we found in other studies that they determined the amount of flavonoid and quercetin concentration. However, No study found icariin in cogon grass extract. This compound is mainly found in horny goat weed (Yin Yang Huo), a Chinese herbal medicine. We added some information about Quercetin (Line 305-307).
